# Suction cup on a piston-based chest compression device improves coronary perfusion pressure and cerebral oxygenation during experimental cardiopulmonary resuscitation

**DOI:** 10.1016/j.resplu.2022.100311

**Published:** 2022-09-29

**Authors:** Johan Mälberg, David Smekal, Silvia Marchesi, Miklós Lipcsey, Sten Rubertsson

**Affiliations:** a Emergency Medical Service (EMS), Uppsala University Hospital, Sjukhusvägen, 75185 Uppsala, Sweden; b Anesthesia and Intensive Care Service, Surgical Science Department Sjukhusvägen, 75185, Uppsala University, Uppsala, Sweden; c Jolife/Stryker AB, Scheelevägen 23633, Lund, Sweden; d Hedenstierna Laboratoriet, Uppsala University Hospital, Sjukhusvägen, 75185 Uppsala, Sweden

**Keywords:** Mechanical chest compression, Coronary perfusion pressure, Cerebral oxygenation, suction cup, Piston-based device, ANOVA, analysis of variance, CerPP, cerebral perfusion pressure, CPP, coronary perfusion pressure, CPR, cardiopulmonary resuscitation, CVP, central venous pressure, EtCO2, end tidal carbon dioxide, FiO2, inspirated fraction of oxygen, ICP, intracranial pressure, NIRS, near infrared spectroscopy, PbtO2, partial pressure of tissue oxygen, PEEP, positive end expiratory pressure, SD, standard deviation, SrO2, cerebral oxygen saturation, ROSC, return to spontaneous circulation, tV, tidal volume, VF, ventricular fibrillation

## Abstract

**Introduction:**

The presented study aimed to investigate whether a mechanical chest compression piston device with a suction cup assisting chest recoil could impact the hemodynamic status when compared to a bare piston during cardiopulmonary resuscitation.

**Methods:**

16 piglets were anesthetized and randomized into 2 groups. After 3 minutes of induced ventricular fibrillation, a LUCAS 3 device was used to perform chest compressions, in one group a suction cup was mounted on the device’s piston, while in the other group, compressions were performed by the bare piston. The device was used in 30:2 mode and the animals were manually ventilated. Endpoints of the study were: end tidal carbon dioxide, coronary and cerebral perfusion pressures, and brain oxygenation (measured using near infrared spectroscopy). At the end of the protocol, the animals that got a return to spontaneous circulation were observed for 60 minutes, then euthanized.

**Results:**

No difference was found in end tidal carbon dioxide or tidal volumes. Coronary perfusion pressure and cerebral oxygenation were higher in the Suction cup group over the entire experiment time, while cerebral perfusion pressure was higher only in the last 5 minutes of CPR. A passive tidal volume (air going in and out the airways during compressions) was detected and found correlated to end tidal carbon dioxide.

**Conclusions:**

The use of a suction cup on a piston-based chest compression device did not increase end tidal carbon dioxide, but it was associated to a higher coronary perfusion pressure.

## Introduction

The use of mechanical chest compression devices has increased over time^1^ and mechanical cardiopulmonary resuscitation (CPR) has been demonstrated to be safe 2, 3, 4, 5, to improve chest compression fraction^6^ and to deliver consistent and high-quality chest compressions in both experimental and clinical studies 7, 8, 9. However, no difference in survival has been associated to its use compared to high-quality manual CPR^10^, ^11^.

Mechanical devices can deliver compressions by a piston compressing the chest or using a thoracic band, squeezing the entire thorax, however piston-based devices better mimic manual chest compressions, the gold standard treatment for cardiac arrest^12^.

Current CPR guidelines underline the importance of a total release of the thorax between compressions to allow cardiac refill and thereby improving cardiac output produced by the compressions^12^. On piston based devices the suction cup is designed to assist the return of the thorax to its neutral position, especially when the elastic recoil of the thorax fails due to thoracic cage deformation^13^.

Active decompression (defined as using a force to pull the chest above its neutral position) has been demonstrated to increase cardiac output and cerebral blood flow in an animal study^14^ and also to increase cerebral oxygenation in a human study^15^. However, the effect of the suction cup assisting the chest back to its neutral position between each compression has never been examined.

The aim of this study was to investigate whether a piston device with a suction cup assisting chest recoil could produce a greater cardiac output and therefore and increase in end tidal carbon dioxide (EtCO_2_) compared to the same piston device without a suction cup.

## Materials and methods

The study was a randomized experimental trial, comparing hemodynamics during mechanical CPR with the LUCAS 3 chest compression device (Jolife AB/Stryker, Lund, Sweden) with and without the suction cup.

The experiments were performed at Hedenstierna Laboratoriet (Uppsala University, Sweden), in accordance with the Arrive 2.0 Guidelines^16^ and the National Research Council’s guidance. Ethical approval was given by the Animal Ethics Board in Uppsala, Sweden (Dnr. 5.8.18-05377/2021).

The animals used were Norwegian Landrance/Yorkshire/Hampshire mixed breed piglets.

### Randomization

At the end of preparation, the pigs were randomized into two groups, one group received chest compressions performed by LUCAS 3 with suction cup (Suction cup group) and the other group received chest compressions performed by LUCAS 3 without suction cup (No-Suction cup group).

A 1:1 randomization was performed using a randomization tool from the Research Randomizer online software.

### Protocol

The animals were anesthetized and placed on a laboratory table (for a detailed description of the anesthesia and preparation protocol, check the supplemental materials, Annex A).

The animals’ thorax was shaved, and defibrillator pads were positioned (latero-lateral positioning, trying to avoid the area where the suction cup would be positioned during resuscitation) and connected to Lifepak 20 (Physio Control, WA, US). Near infra-red spectroscopy (NIRS) sensors were placed on the animals’ forehead (the pads were placed 5–6 mm medially to edge of the animal’s orbital cavity on both sides; the skin was carefully shaved and cleaned in alcoholic solution) and Edwards Foresight Elite monitor (Edwards Lifescience, CA, USA) was activated to check the quality of the signal.

A neck artery was isolated, and a catheter was positioned in the aorta (the catheter was advanced 10 to 15 cm, till reaching the ascending tract of the aortic arch) for aortic pressure measurement and on the same side the internal jugular vein was catheterized for measurement of the central venous pressure (considered as a proxy for right atrial pressure). Both catheters were fluid filled, and used for blood gas samples collection, as well.

The cranial parietal region (left or right) was shaved; a small dissection was performed to expose the parietal bone, and a hole was drilled in the cranium to place an intra-cranial pressure (ICP) and Brain Tissue Partial oxygen pressure (PbtO_2_) sensor in the animals’ brain. The ICP sensor was connected to the monitor and the PbtO_2_ sensor were connected to Licox CMP machine (GMS, Germany). The PbtO_2_ sensor was allowed to stabilize for 20–30 minutes before starting to take measurements.

The device was positioned on the animals’ chest. Both the device and the suction cup used during experiments were the same as the ones used on patients. In the Suction cup group, the suction cup’s brim was glued to the thorax to get full attachment (the glue was used because of the pointier chest of the pigs which makes it difficult to maintain the vacuum inside the suction cup compared to what happens during use in humans). In the No-Suction cup group, the compression was performed directly by the piston pad (the suction cup was not mounted on the device).

The hooves of the animals were fixated to the surgical table with thick tape to assure stillness during the experiment; besides, sandbags were placed to fill the space between the animals’ flanks and the device legs.

Two needles were placed in the subcutaneous tissue on the chest, creating a line passing over the pig’s heart. A device induced ventricular fibrillation (VF) by delivering an alternating current (80 V – 500 VA). VF was verified by the disappearance of the aortic pressure and ECG curve.

Three minutes of untreated VF preceded the start of CPR. The device was used in 30:2 mode (with a three-second ventilation pause after every 30 compressions) and it was programmed so that the piston came back to the starting position after every compression in both groups.

The position of the piston on the chest was not readjusted during the experiment in any of the groups. To ensure piston displacement’s detection, a mark was drawn with a surgical skin marker on the compression point, so that the assessment of the position stability was easier.

During CPR, ventilation was performed manually and by the same person for all the experiments. Animals remained connected to the respiratory circuit and a bag valve was used to deliver breaths. FiO_2_ during CPR was set to 1.0.

An adrenalin bolus of 0.5 mg was administered after 18 minutes.

After 20 minutes from CPR’s start, defibrillation was performed with a 150 J biphasic impulse. CPR was then resume for a 2-minutes cycle followed by another defibrillation. If no return to spontaneous circulation (ROSC) was detected after 2 cycles (and a total of 3 shocks) or if the animal was found with a non-shockable rhythm, the animal was declared dead. In case of ROSC the animal was observed and monitored for 60 minutes, and arterial and venous blood gas were performed after 30 minutes. At the end of the 60 minutes of observation, animals were euthanized by the administration of potassium chloride.

Autopsy was performed to assess CPR-related injuries by the researcher not blinded to the protocol (the autopsy protocol is presented in the Supplemental material – Annex B).

**Fig. 1:**
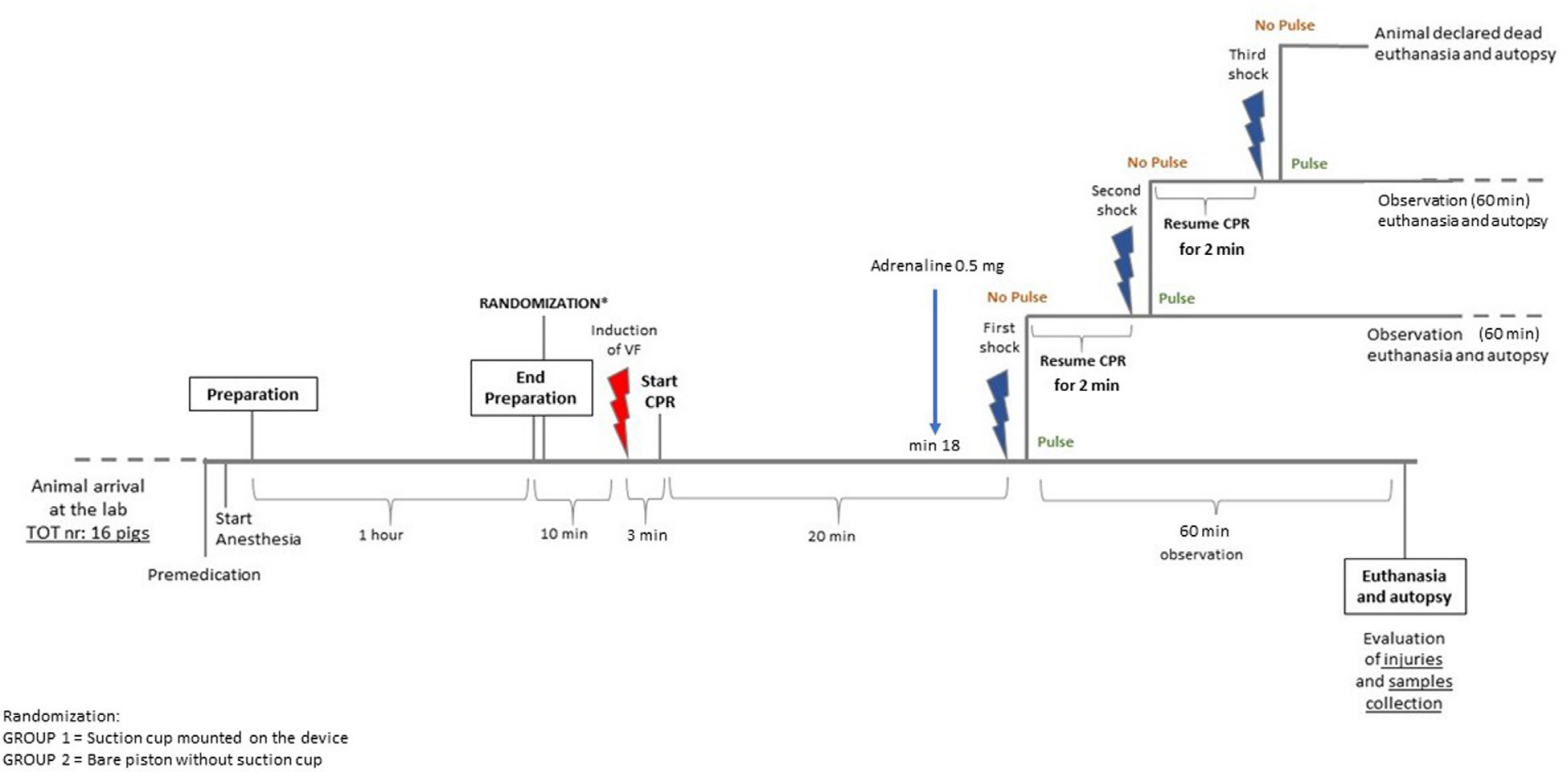
**Trial flowchart.** The details of the trial protocol are presented on a timeline, in the flowchart.

A flowchart of the protocol is shown in Fig. 1.

### Endpoints and other measurements

Primary endpoints:

• End tidal carbon dioxide (EtCO_2_) - mmHg;

Secondary endpoints:

• Coronary perfusion pressure (CPP) (calculated from aortic and venous pressure at the end of the decompression phase) - mmHg;
• Cerebral perfusion pressure (CerPP) (calculated from aortic pressure and ICP at the end of the decompression phase) - mmHg;
• Compression and decompression (or peak and nadir) aortic pressure - mmHg;
• Cerebral oxygenation (SrO_2_) - %;
• Cerebral tissue oxygen partial pressure (PbtO_2_) – mmHg.

Other measurements:

• Central venous pressure (CVP) – mmHg;
• Intracranial pressure (ICP) – mmHg;
• tidal volume during CPR – ml;
• injuries assessment;
• the number of adverse events and device adverse events;
• chest height (at the beginning and at the end of the CPR).

Aortic, central venous (CVP) and intracranial pressure (ICP) were acquired 100 times per second, while one value of EtCO_2_ and tidal volume (tV) were obtained at each breath.

To analyse hemodynamic parameters, EtCO_2_ and cerebral oxygenation data (SrO_2_ and PbtO_2_), the CPR time (20 minutes in total) was divided into 4 timeframes: from 0 to 5 minutes, from 5 to 10 minutes, from 10 to 15 minutes and from 15 to 20 minutes. The average value of all the variables was calculated for the four intervals.

The aortic, CVP and ICP pressure data were analysed in three different points of the curve at every compression/decompression cycle: *peak pressure*, *nadir pressure* and at the end of the decompression phase (*End of decompression pressure*). The *End of decompression pressure* was used to calculate coronary perfusion pressure (CPP) and cerebral perfusion pressure (CerPP)^14^.

CPP = Aortic pressure – CVP (at the end of the decompression phase).
CerPP = Aortic pressure – ICP (at the end of decompression phase).

A ratio dividing the mean value of SrO_2_ and PbtO_2_ for every timeframe with their baseline values (SrO_2_/SrO_2 baseline_; PbtO_2_/PbtO_2 baseline_) were obtained and use for analysis.

### Statistical analysis

Data analysis was performed using Prism 8.0 software. According to sample size calculation (α error 0.05, β error 20% to detect a 5 ± 1.6 mmHg difference in EtCO_2_), 16 piglets were included in the study, 8 per group.

Data normal distribution was verified with the Shapiro-Wilk test. Parametric data are presented as mean and standard deviation (SD) in tables, text, and graphs, while non-parametric data are represented as median and range (lowest and highest value).

The comparison between the groups was performed using the mixed model ANOVA for repeated measures data and multiple t-test for parametric data (such as hemodynamics, respiratory and neurological data), and with Wilcoxon test for non-parametric data (injuries rate and anterior-posterior chest height). Pearson’s test was used to investigate correlation between variables; correlation coefficient with 95% CI (confidence interval) and r^2^ are presented.

A *p value* < 0.05 was considered significant.

## Results

Sixteen piglets between 2 and 3 months old were included in the presented study. Eight were randomized in the Suction cup group and 8 in the No-Suction cup group. Average weight was 30.5 (27.2 – 32.8) kg, and 2 out of 16 piglets were male, while the rest of the animals were female (both male piglets were randomized in the No-Suction cup group).

Hemodynamic and respiratory parameters at baseline where similar between the groups (reported in Table 1A – Supplementary material, Annex C).

**Table 1:** Aortic pressure (AP), central venous pressure (CVP) and intracranial pressure (ICP), measured at the peak, nadir and at the end of the decompression phase (mean and standard deviation) for every timeframe during CPR. Difference between the groups calculated with multiple comparison (correction methods: Bonferroni-Dunn) was represented as a * (p < 0.05) or a ** (p < 0.01) in the Suction cup group cell.

	0–5 min			5–10 min			10–15 min			15–20 min		
	Suction cup	No suction cup	*p value*	Suction cup	No suction cup	*p value*	Suction cup	No suction cup	*p value*	Suction cup	No suction cup	*p value*
Peak AP (mmHg)	119 ± 63	94 ± 32	*0.4*	100 ± 48	81 ± 25	*0.38*	92 ± 44	74 ± 23	*0.42*	85 ± 42	74 ± 29	*0.62*
Nadir AP (mmHg)	11 ± 9	1 ± 14	*0.13*	10 ± 9	2 ± 10	*0.21*	8 ± 8	1 ± 11	*0.22*	8 ± 8	5 ± 10	*0.54*
End of decompresAP(mmHg)	31 ± 4	27 ± 4	*0.04* *	25 ± 9	23 ± 5	*0.69*	23 ± 9	22 ± 6	*0.77*	23 ± 8	21 ± 6	*0.75*
Peak CVP (mmHg)	85 ± 96	126 ± 67	*0.42*	65 ± 67	104 ± 47	*0.27*	60 ± 60	95 ± 39	*0.25*	53 ± 49	86 ± 35	*0.21*
Nadir CVP (mmHg)	2 ± 4	6 ± 2	*0.02* *	0.3 ± 4	6 ± 2	*0.01* *	−2 ± 4	6 ± 2	*0.003* **	3 ± 3	7 ± 2	*0.01* *
End of decompresCVP(mmHg)	10 ± 4	15 ± 2	*0.06*	10 ± 5	13 ± 6	*0.48*	10 ± 5	12 ± 6	*0.47*	9 ± 6	13 ± 6	*0.31*
Peak ICP (mmHg)	31 ± 5	26.7 ± 5	*0.8*	26 ± 4	24 ± 4	*0.56*	24 ± 15	23 ± 16	*0.89*	23 ± 18	22 ± 8	*0.71*
Nadir ICP (mmHg)	8 ± 5	8 ± 4	*0.95*	6 ± 5	6 ± 7	*0.9*	5 ± 4	8 ± 2	*0.06*	6 ± 5	9 ± 4	*0.28*
End of decompres ICP(mmHg)	10 ± 5	10 ± 4	*0.63*	8 ± 5	11 ± 4	*0.38*	8 ± 5	11 ± 3	*0.2*	8 ± 5	11 ± 3	*0.19*

### EtCO_2_ and respiratory parameters

**Fig. 2:**
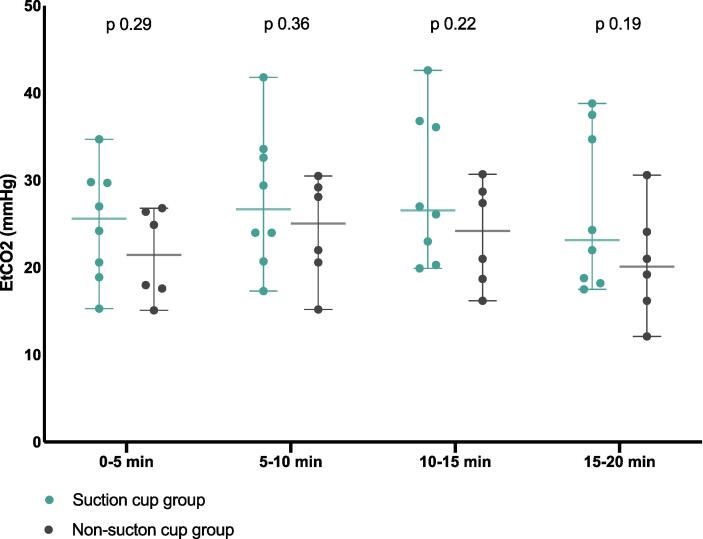
**EtCO_2_ vs timeframe in the two groups.** Single animals’ values are represented as dots. Middle line represents mean and peripheral lines 95% CI (confidence interval). EtCO_2_ is measured in mmHg (millimetres of mercury). EtCO_2_: end tidal carbon dioxide.

EtCO_2_ value was measured in 14 animals out of 16, as in two animals it was impossible due to technical problems with the data collection system. No difference was found in the tV (tidal volume) delivered manually to the two groups (mean (SD): 294 ml (9.6 ml/kg) for the Suction cup group and of 282 ml (9.1 ml/kg) for the No-Suction cup group); and there was no difference in EtCO_2_ between the groups (Fig. 2).

The ventilator detected a passive movement of air during compressions (passive tV), which represented the volume of air going in and out the airways at every compression.

Passive tV was found similar in both groups (mean value: 40 ml/compression in the Suction cup group and 34 ml/compression in the No-Suction cup group).

**Fig. 3:**
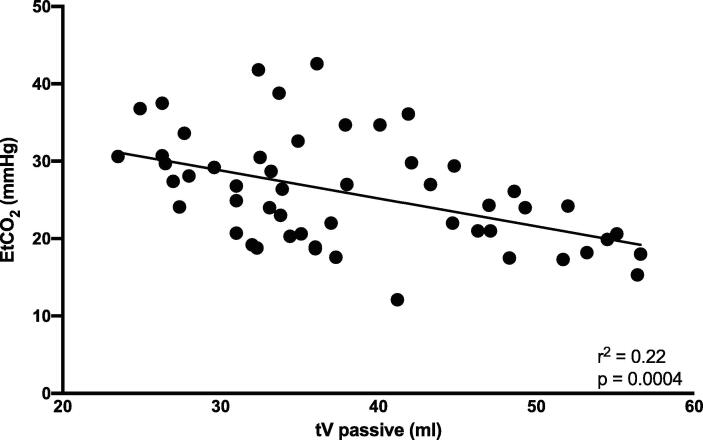
**EtCO2 vs tV passive.** The dots represent single animals’ values of EtCO2 vs passive tV at different time points. Correlation line is presented. tV passive = passive tidal volume (volume of air going in and out of the airways at every compression/decompression). EtCO_2_: end tidal carbon dioxide; mmHg: millimetres of mercury; ml: milliliters.

There was no correlation between EtCO_2_ and hemodynamic measurements, tV or minute volume ventilation, but there was a negative correlation between EtCO2 and passive tV (correlation coefficient: −0.39 [95%CI −0.55, −0.16]. r^2^ 0.22; p 0.004) (Fig. 3).

### Hemodynamic data

Baseline values are presented in Table1A (Supplementary material, Annex C). No statistically significant difference was identified between the groups.

Aortic, central venous and intra-cranial pressure measurements during CPR are shown in Table 1.

At the end of decompression, aortic pressure was higher in the Suction cup group at the beginning of the CPR (Timeframe 1: from 0 to 5 minutes).

Nadir CVP was found lower in the Suction cup group by mixed effect ANOVA and at all timeframes by multiple t-tests.

CPP was found higher in the Suction cup group by mixed effect ANOVA and at timeframe 1 (0–5 min), 2 (5–10 min) and 4 (15–20 min) by multiple t-tests.

**Fig. 4:**
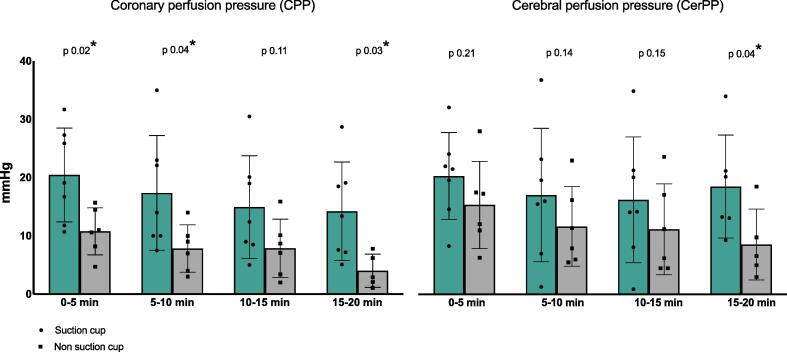
**Coronary perfusion pressure (CPP) and cerebral perfusion pressure (CerPP).** CPP and CerPP mean values (represented by the histogram) and standard deviation (ST) (represented by the error line). Individual value are also reported as dots or squares. *p value* for groups comparison (t-test with Bonferroni-Dunn correction) is reported over the correspondent timeframe space. *p value* < 0.05 were considered significant and are indicated by a *. CPP and CerPP were measured in mmHg (millimetres of mercury).

CerPP was higher in the Suction cup group at timeframe 4 (15–20 min) by multiple t-tests (Fig. 4).

Both variables significantly decreased overtime.

### Cerebral oxygenation

**Fig. 5:**
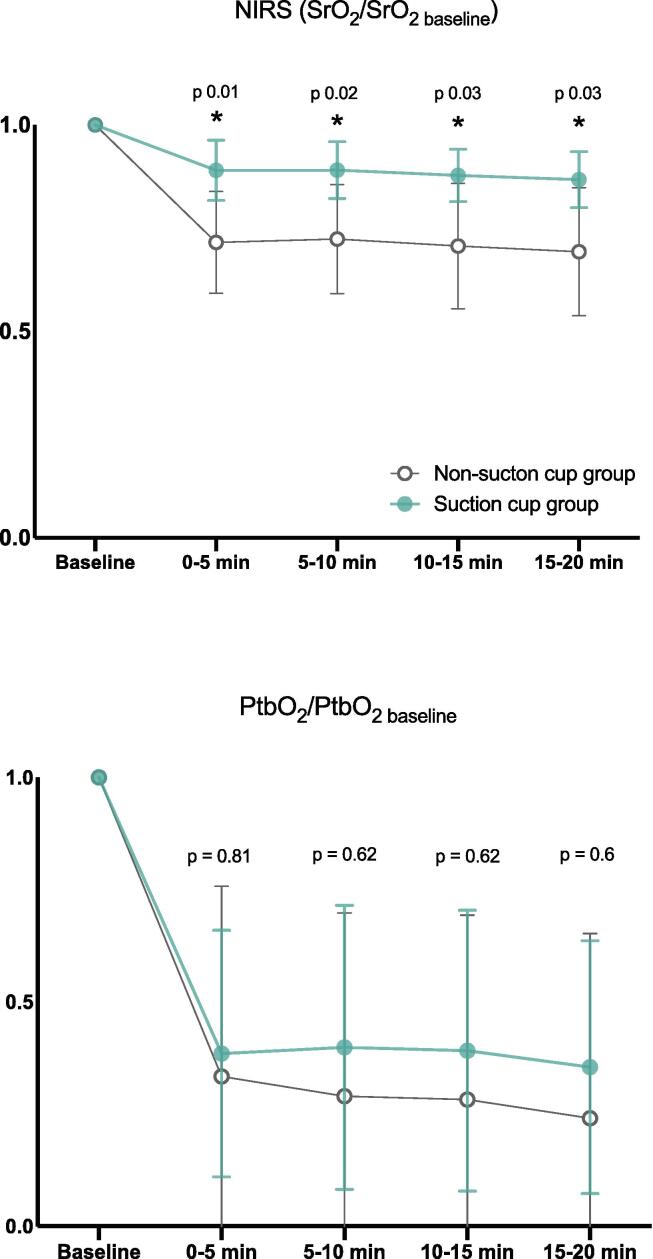
**SrO_2_ and PtbO_2_ data.** The mean value of SrO_2_ (cerebral oxygen saturation) and PtbO_2_ (cerebral tissue partial pressure of oxygen) for each timeframe divided by their value at baseline is represented by a dot. The error lines represent standard deviation. *p value* for groups comparison (t-test with Bonferroni-Dunn correction) reported over the correspondent timeframe space. *p value* < 0.05 were considered significant and are indicated by a *. NIRS: near infrared spectroscopy.

SrO_2_/SrO_2 baseline_ ratio was higher in the Suction cup group compared to the No-Suction cup group at every timeframe (Fig. 5), while ptbO_2_/ptbO_2 baseline_ ratio was found similar at every timeframe (Fig. 5).

The values of SrO2 and ptbO2 at baseline and at each timeframe in the two groups are reported in the Supplemental material (Annex D, Table 2A and 3A).

### Injuries

The only injuries detected during the autopsies were: skin wounds (light bruises), rib fractures and lung contusion (mainly in the paracardiac and basal area).

No animals had sternal fractures or other visceral injuries.

The occurrence of skin wounds was similar between the groups.

The median number of right rib fractures were 0 in the Suction cup group and 1 in the No-Suction cup group. For left rib fractures the median number was 3.5 in the Suction cup group and 3 in the No-Suction cup group.

Two animals in the Suction cup group and 1 in the No-Suction cup group presented diffuse lung contusion. All the others presented a moderate contusion concentrated in the lower-medial part of the lungs.

Three animals got ROSC and two of them survived 60 minutes in the Suction cup group, while one animal survived in the No-Suction cup group. The survival rate is reported as an additional information, but no statistical analysis has been performed as the sample size was too small to conduct that kind of calculations.

### LUCAS 3 data

No device failures and no suction cup detachment were reported. The piston position in the No suction cup group, assessed visually by comparing the point touched by the piston with the skin mark drawn before the protocol started, was stable in all the animals.

Compression depth and rate were the same in both groups.

The chest height before the CPR started was 21 cm (17.5 – 22.5) (median and range). The difference in Anterior Posterior diameter created by the chest collapse was similar in the two group and was 1.6 (0.7 – 3) cm in the Suction cup group and 2 (1 – 2.8) cm in the No-Suction cup group.

## Discussion

In the presented experimental trial comparing piston-based mechanical CPR with and without the use of a suction cup, no difference in EtCO_2_ was detected.

Otherwise, the coronary perfusion pressure (CPP) was significantly higher when the suction cup was used. This difference was observed throughout the CPR time, although in both groups CPP decreased overtime. Interestingly nadir central venous pressure (the lowest value of CVP over a cycle) was lower in the Suction cup group at all timeframes and it could be speculated that a lower minimum pressure in the right atrium would improve the heart refilling, consequently increasing cardiac output, which could explain the better CPP.

The lack of statistical difference in EtCO_2_ associated with an increase in the coronary perfusion pressure, can be explained by the small number of animals studied, considering that, for technical reasons, only 6 animals were included in the analysis on EtCO_2_ in the No suction cup group. Besides, the increase of the passive ventilation, the amount of air moving in and out the airways during compressions and recoils/decompressions, as demonstrated by Shultz et al^17^, can influence EtCO_2_. Even if the value of passive ventilation was not found significantly different, the value was numerically higher during the suction cup use. EtCO_2_ correlated to passive tidal volume, but no other parameters (including the tidal volume of hand-bag ventilation, measured as a whole and as ml/kg of weight). Based on this, when assessing EtCO_2_ one may need to consider the effect of passive ventilation, especially in experimental settings when comparing different chest compression technique. To date, further studies are needed to confirm this hypothesis.

In accordance with the higher CPP, cerebral oxygenation saturation (calculated as a ratio to the baseline value) was found higher in the Suction cup group, even if an associated increase in tissue oxygen partial pressure could not be identified. These results are to some extent in line with a previous experimental study showing a higher cerebral flow produced in the group receiving mechanical chest compressions with the device including a suction cup versus a manual sternal compressor (Cardiopress, Resuscitation Laboratories, Bridgeport, CT, USA). This device was used to get a standardised depth of the manual chest compressions^18^.

As this is the first study investigating the effect of the lifting of anterior chest wall to the thorax neutral position as a component of mechanical CPR able to facilitate a full recoil at every decompression, it is not possible to compare our results with other data. Otherwise, previous studies investigated the effect of active decompression, both on a similar animal model and in clinical trials. Active decompression is defined as exerting a certain force in the decompression phase, thus lifting the chest above its natural position of rest. Experimental studies, investigating the effect of active decompression on pigs’ hemodynamics, found a higher cerebral blood flow and cardiac output compared with a non-active decompression approach^14^, ^19^, ^20^. A human study comparing a modified LUCAS 2 device with an active decompression feature vs a standard LUCAS 2 device found a better cerebral oxygen saturation when the active decompression was correctly administered^15^.

Even if CPR-related chest collapse has not been measured in humans before, the changes in chest compliance during CPR have been demonstrated by a previous cadaver study: the elastic recoil of the human thorax decreases over time^13^, which could negatively affect the heart refilling. In this investigation the Anterior Posterior chest diameter difference of roughly 1.5 cm between the start and the end of CPR represents the chest collapse produced by CPR. The potential benefit of the suction cup seems to increase over time possibly related to the progressive decrease in the thoracic compliance.

It is important to underline that in pigs, due to thoracic anatomical differences compared to humans (high and pointy chest), the thoracic collapse during CPR may be more pronounced and the effect of the suction cup lifting the chest back to the starting position could have been magnified by the animals’ anatomical specificity.

During the study, the device (with or without suction cup) was not re-adjusted to the progressive reduction of Anterior Posterior diameter, even if, due to the chest collapse, a gap between the piston and the thorax occurred during decompressions in the No-Suction cup group. The decision for a non-readjustment strategy was taken to avoid deeper compressions and increased rib cage and visceral injuries, which would have affected the results of the study.

From a safety perspective, the use of the suction cup did not influence the rate or type of injuries.

### Limitations

The presented study has some potential limitations. Firstly, although this is a relevant pre-clinical animal study, the results cannot be transferable directly to the clinical settings. The pig’s chest anatomy differs from the human chest in shape and height, and this could have brought to results difficult to replicate in humans, as discussed above. Otherwise, the porcine model is a well-established one and used for research on cardiopulmonary resuscitation with results often reproducible in humans.

Secondly, NIRS has been previously used in pigs, but the thicker skin and skull of the animals interfere with the establishment of an adequate signal, and it might be difficult to assess if measurements refer to the brain tissue or to subcutaneous tissues or muscular; baseline values result in most of the cases lower than what measured in humans, that is the reason why the analysis of NIRS data are presented as a ratio with the baseline measurement. Otherwise, NIRS has been used in pigs in the past^21^.

Lastly, the CPR time in the experiments was limited to 20 minutes, the effect of suction cup for longer resuscitations has not been investigated.

## Conclusions

The application of a suction cup on a piston chest compression device, in this swine cardiac arrest model did not increase EtCO_2_, but it was associated to a higher coronary perfusion pressure. Due to the small numbers of animals in this study, further studies are needed to elucidate eventual survival benefits related to the suction cup.

## CRediT authorship contribution statement

**Johan Mälberg:** Conceptualization, Formal analysis, Investigation, Data curation, Writing – original draft, Writing – review & editing. **David Smekal:** Conceptualization, Methodology, Validation, Supervision, Writing – review & editing. **Silvia Marchesi:** Conceptualization, Investigation, Visualization, Writing – original draft. **Miklós Lipcsey:** Conceptualization, Validation, Writing – review & editing. **Sten Rubertsson:** Conceptualization, Methodology, Validation, Supervision, Writing – review & editing.

## Declaration of Competing Interest

The authors declare the following financial interests/personal relationships which may be considered as potential competing interests: [The authors declare that part of the financial support for the presented research was provided by Stryker; Stryker being the company manufacturing the mechanical chest compression device used in the study. Silvia Marchesi declares that she was employed by Stryker during the time she worked on the research.]

## References

[1] Kahn P.A., Dhruva S.S., Rhee T.G., Ross J.S. (2019). Use of Mechanical Cardiopulmonary Resuscitation Devices for Out-of-Hospital Cardiac Arrest, 2010–2016. JAMA Netw Open.

[2] Koster R.W., Beenen L.F., van der Boom E.B. (2017). Safety of mechanical chest compression devices AutoPulse and LUCAS in cardiac arrest: a randomized clinical trial for non-inferiority. Eur Heart J.

[3] Smekal D., Lindgren E., Sandler H., Johansson J., Rubertsson S. (2014). CPR-related injuries after manual or mechanical chest compressions with the LUCAS^TM^ device: A multicentre study of victims after unsuccessful resuscitation. Resuscitation.

[4] Smekal D., Hansen T., Sandler H., Rubertsson S. (2013). Comparison of computed tomography and autopsy in detection of injuries after unsuccessful cardiopulmonary resuscitation. Resuscitation.

[5] Smekal D., Johansson J., Huzevka T., Rubertsson S. (2009). No difference in autopsy detected injuries in cardiac arrest patients treated with manual chest compressions compared with mechanical compressions with the LUCAS^TM^ device-A pilot study. Resuscitation.

[6] Olasveengen T.M., Wik L., Steen P.A. (2008). Quality of cardiopulmonary resuscitation before and during transport in out-of-hospital cardiac arrest. Resuscitation.

[7] Esibov A., Banville I., Chapman F.W., Boomars R., Box M., Rubertsson S. (2015). Mechanical chest compressions improved aspects of CPR in the LINC trial. Resuscitation.

[8] Tranberg T., Lassen J.F., Kaltoft A.K. (2015). Quality of cardiopulmonary resuscitation in out-of-hospital cardiac arrest before and after introduction of a mechanical chest compression device, LUCAS-2; a prospective, observational study. Scand J Trauma Resusc Emerg Med.

[9] Gyory R.A., Buchle S.E., Rodgers D., Lubin J.S. (2017). The efficacy of lucas in prehospital cardiac arrest scenarios: A crossover mannequin study. West J Emerg Med.

[10] Brooks S.C., Bigham B.L., Morrison L.J. (2011). Mechanical versus manual chest compressions for cardiac arrest. Cochrane Database Syst Rev.

[11] Wik L., Olsen J.A., Persse D. (2014). Manual vs. integrated automatic load-distributing band CPR with equal survival after out of hospital cardiac arrest. The randomized CIRC trial. Resuscitation.

[12] Panchal AR, Bartos JA, Cabañas JG, et al. *Part 3: Adult Basic and Advanced Life Support: 2020 American Heart Association Guidelines for Cardiopulmonary Resuscitation and Emergency Cardiovascular Care*. Vol 142.; 2020. 10.1161/CIR.0000000000000916. 33081529

[13] Segal N., Robinson A.E., Berger P.S. (2017). Chest compliance is altered by static compression and decompression as revealed by changes in anteroposterior chest height during CPR using the ResQPUMP in a human cadaver model. Resuscitation.

[14] Steinberg M.T., Olsen J.A., Eriksen M. (2018). Haemodynamic outcomes during piston-based mechanical CPR with or without active decompression in a porcine model of cardiac arrest. Scand J Trauma Resusc Emerg Med.

[15] Berve P.O., Hardig B.M., Skålhegg T., Kongsgaard H., Kramer-Johansen J., Wik L. (2021). Mechanical active compression-decompression versus standard mechanical cardiopulmonary resuscitation: A randomised haemodynamic out-of-hospital cardiac arrest study. Resuscitation.

[16] du Sert N.P., Hurst V., Ahluwalia A. (2020). The arrive guidelines 2.0: Updated guidelines for reporting animal research. PLoS Biol.

[17] Shultz J.J., Coffeen P., Sweeney M. (1994). Evaluation of standard and active compression-decompression CPR in an acute human model of ventricular fibrillation. Circulation.

[18] Rubertsson S., Karlsten R. (2005). Increased cortical cerebral blood flow with LUCAS; a new device for mechanical chest compressions compared to standard external compressions during experimental cardiopulmonary resuscitation. Resuscitation.

[19] Moore J.C., Holley J., Segal N. (2018). Consistent head up cardiopulmonary resuscitation haemodynamics are observed across porcine and human cadaver translational models. Resuscitation.

[20] Ryu H.H., Moore J.C., Yannopoulos D. (2016). The Effect of Head Up Cardiopulmonary Resuscitation on Cerebral and Systemic Hemodynamics. Resuscitation.

[21] García-Bardon A., Kamuf J., Ziebart A. (2021). Levosimendan increases brain tissue oxygen levels after cardiopulmonary resuscitation independent of cardiac function and cerebral perfusion. Sci Rep.

