# Supplementary material for: Suction cup on a piston-based chest compression device improves coronary perfusion pressure and cerebral oxygenation during experimental cardiopulmonary resuscitation
Source: Resusc Plus. 2022 Sep 29;12:100311. doi: 10.1016/j.resplu.2022.100311 (PMC9525897; doi:10.1016/j.resplu.2022.100311)
Supplement: Supplementary Data 1 [file mmc1.docx]

# Supplementary material

## ANNEX A

## Anesthesia and preparation

The animals were transported directly from the farmer to the lab and were weighed upon arrival. They fasted in the previous 12 hours, with free access to water.

Premedication was an intramuscular (i.m.) injection of tiletamine and zolazepam (6 mg Kg^-1^) and xylazine (2.2 mg Kg^-1^). After 5 to 10 minutes, the animals were placed supine on an operating table; an ear vein was cannulated to administer a bolus of fentanyl (10-20 mcg Kg^-1^). A peripheral oxygen saturation probe was placed on the animals’ tail. Tracheotomy was performed and a 7 mm internal diameter (ID) tube was positioned. ECG electrodes were positioned and connected to the monitor.

Deep anaesthesia was maintained with a continuous infusion of ketamine, midazolam and fentanyl. After checking that anaesthesia was enough to prevent responses to painful stimulation, a bolus of muscle relaxant (rocuronium 50 mg) was administered followed by a continuous infusion (0.15 mg/kg/h), to avoid gasping during the experiment. In case of ROSC, the administration of Rocuronium was interrupted.

Ringer Acetate was administered (30 ml/kg/h) during preparation to reduce the risk of hypovolemia. After the end of preparation, the administration of fluids was discontinued.

During the preparation phase the animals were ventilated in volume control mode with the following parameters: tV (tidal volume) 6-8 ml/kg, respiratory rate 20, inspiratory: expiratory ratio 1:2, FiO_2_ (fraction of inspired oxygen) 0.4, PEEP (positive end expiratory pressure) 5 cmH_2_O.

## ANNEX B.

## Post-mortem examination

### Euthanasia protocol

At the end of the experiment, animals are euthanized with a KCl injection (20 ml injected in the central venous line – around 85 mg/kg or a total of 70 mEq). In case the animal does not get ROSC (return to spontaneous circulation) at the end of CPR, the KCl injection is administered, and chest compressions continued for 20 to 30 seconds to get the euthanasia drug to the animal heart before starting the autopsy.

Asystole was verified before proceeding.

### Autopsy protocol

A. External observation.

- Assessment of possible airways bleeding.

- The endotracheal tube is inspected, then aspiration is performed inside the tube before extubating the animal.

- Assessment of:

1. the amount of blood present on the aspiration tube is reported as: ***none, few stripes, pink foam, moderate stripes, big amount of blood and clots***.

2. The amount of blood in the endotracheal tube at extubating (quantified from **1** to **3**).

- Assessment of the thoracic skin (on the piston/suction cup covered area) and description of cutaneous damage using one of the following: ***none, light bruise, dark bruise, subcutaneous/cutaneous haemorrhage, scraping/abrasion***.
- Check the presence of subcutaneous emphysema (***yes/no***)

B. Internal assessment.

After an external check the thorax and abdomen of the animals are surgically opened exposing thorax and abdomen:

- The pleural space is checked for emo- and pneumothorax (the amount of blood is specified if possible);
- Sternal fracture (***none, complete, incomplete, multiple***) and costal fracture (***how many and on which side***) are reported;
- Big vessels integrity is reported;
- Lungs and heart are removed in-block from the rib cage dissecting vessel and trachea. Definition of lung damage: ***none, contusion, laceration*** (and the extension).

Definition of heart damage: ***none, contusion, laceration, rupture***;

- Observation of liver and spleen, report injury by specifying ***none, contusion, laceration*** (and how much blood is detected in case of laceration, if possible).
- Observe the rest of the abdomen and report any other injuries (intestinal laceration, other haemorrhages).

| **Autopsy findings form** | | | | | | |
| --- | --- | --- | --- | --- | --- | --- |
| External Evaluation | | | | | | |
| **Thoracic skin** | None | Light bruise | Dark bruise | | Subcut/cut. haemorrhage | Scarping/abrasion |
| **Airways bleeding**  (aspiration) | None | Few stripes | Pink foam | | Moderate stripes | Big amount and clots |
| **Airways bleeding**  (on the tube after extubation) | None | 1 | 2 | | 3 |  |
| **Subcutaneous emphysema** | YES | | | | NO | |
| Internal Evaluation | | | | | | |
| **Pleural space** | Haemothorax  Right | Haemothorax Left | Pneumothorax  Right | | Pneumothorax Left | None |
| **Sternal fracture** | None | Complete | Incomplete | | Multiple | |
| **Rib fracture**  (indicate number) | Right | | | | Left | |
| **Big vessels**  (especially vena cava) | None | | | | Damage (explain) | |
| **Lungs** | None | Contusion | | Laceration | | |
| **Heart** | None | Contusion | Laceration | | Rupture | |
| **Liver** | None | Contusion | | | Laceration | |
| **Spleen** | None | Contusion | | | Laceration | |
| **Abdominal haemorrhage** | Yes (indicate quantity if possible) | | | | No | |
| **Others** |  | | | | | |

Figure B1. Autospy report (to fill in).

## ANNEX C.

**Table 1A.** Baseline hemodynamic parameters per group (mean and standard deviation – SD); abbreviations: SpO_2_, peripheral saturation of oxygen; EtCO_2_, end-tidal carbon dioxide; bpm, beat per minute; mmHg, millimetres of mercury.

|  | **Suction cup group** | | **No suction cup group** | |
| --- | --- | --- | --- | --- |
|  | Mean | SD | Mean | SD |
| Pulse (bpm) | 69 | 5 | 80 | 16 |
| Systolic arterial pressure  (SAP; mmHg) | 106 | 14 | 116 | 15 |
| Mean arterial pressure (MAP;mmHg) | 85 | 15 | 98 | 15 |
| Diastolic arterial pressure (DAP; mmHg) | 71 | 14 | 82 | 14 |
| Central venous pressure (CVP; mmHg) | 9 | 2 | 9 | 3 |
| SpO_2_ (%) | 100 | 0 | 100 | 0 |
| EtCO_2_ (mmHg) | 4,6 | 0,6 | 4,8 | 0,8 |
| Intra-cranial pressure  (ICP; mmHg) | 10 | 5 | 10 | 3 |

## ANNEX D.

### SrO_2_ and PbtO_2_ (average value ± standard deviation per group per timeframe)

#### Table 2A. SrO_2_

|  | Baseline | 0-5 min | 5-10 min | 10-15 min | 15-20 min |
| --- | --- | --- | --- | --- | --- |
| Suction cup group | 47 ± 8 | 42 ± 9 | 42 ± 10 | 41 ± 10 | 41 ± 9 |
| No suction cup group | 49 ± 11 | 37 ± 10 | 38 ± 11 | 28 ± 8 | 37 ± 10 |

#### Table 3A. PbtO_2_

|  | Baseline | 0-5 min | 5-10 min | 10-15 min | 15-20 min |
| --- | --- | --- | --- | --- | --- |
| Suction cup group | 45 ± 5 | 13 ± 11 | 15 ± 10 | 15 ± 9 | 12 ± 10 |
| No suction cup group | 52 ± 23 | 15 ± 13 | 13 ± 12 | 13 ± 12 | 11 ± 10 |
